# Comment on Casiraghi et al. “Mucoadhesive Budesonide Formulation for the Treatment of Eosinophilic Esophagitis” 2020, *12*, 211

**DOI:** 10.3390/pharmaceutics12090829

**Published:** 2020-08-31

**Authors:** Eyal Zur

**Affiliations:** Pharmaceutical Compounding Consultancy Agency: “Compounding Solutions”, Tel-Mond 4062269, Israel; eyal@eyalzur-pharm.co.il or eyalzur62@gmail.com; Tel.: +972-506-561-982

**Keywords:** eosinophilic esophagitis, oral viscous budesonide, compounding, compounding pharmacist

## Abstract

In their article, Casiraghi, A. et al. describe a few relevant methods to assess the quality of a pharmaceutical preparation of oral viscous budesonide, intended to be swallowed, and treat the esophagus in eosinophilic esophagitis patients. They choose the following methods for this purpose: rheological properties, syringeability, mucoadhesiveness, and in vitro penetration of budesonide in porcine esophageal tissue. At the end of the article, they concluded that the best formulation of oral viscous budesonide was the one already being used in hospitals, based on xanthan gum. In their article, the authors did not emphasize that this specific formula was developed by the compounding pharmacist Eyal Zur from Israel and was published eight years before, as part of an article in the International Journal of Pharmaceutical Compounding. The purpose of this comment is to give the appropriate credit to the pharmacist who first developed and published this well designed formulation.

## 1. Introduction

Oral viscous budenoside is the drug of choice for the treatment of eosinophilic esophagitis in adults and children world-wide. Compounding pharmacists supply the demand for this medication as, in most of the world, no commercial, industrial medication is available.

## 2. Discussion

In their article, Casiraghi, A. et al. claim that [1] “the compounding procedures (in order to compound oral viscous budesonide-author’s addition) are empirical and the composition is not supported by real physicochemical and technological characterization”. Indeed, Casiraghi et al. analyzed a few relevant aspects of the formulation of oral viscous budesonide in their article, such as rheological properties, syringeability, mucoadhesiveness, and in vitro penetration of budesonide in porcine esophageal tissue. At the end of the article, Casiraghi, A. et al. concluded that the best formulation of oral viscous budesonide was the one already being used in hospitals, based on xanthan gum. In their article, the authors did not emphasize that this specific formula was developed by the compounding pharmacist Eyal Zur from Israel, and was published eight years before [2]. The only difference in the formulas related to one of the two sweeteners that was used.

## 3. Conclusions

The formula Casiraghi, A. et al. presented and concluded was the “best” was my formula, and there is no credit given to the formula, nor was there a discussion of the fact that it had been developed and published eight years prior to their publication [2].
